# Role of the Intramural Vascular Network of the Extrahepatic Bile Duct for the Blood Circulation in the Recipient Extrahepatic Bile Duct Used for Duct-to-Duct-Biliary-Anastomosis in Living Donor Liver Transplantation

**DOI:** 10.3389/ti.2022.10276

**Published:** 2022-05-03

**Authors:** Naotaka Yamaguchi, Ryusei Matsuyama, Yutaro Kikuchi, Sho Sato, Yasuhiro Yabushita, Yu Sawada, Yuki Homma, Takafumi Kumamoto, Kazuhisa Takeda, Daisuke Morioka, Itaru Endo, Hiroshi Shimada

**Affiliations:** Department of Gastroenterological Surgery, Graduate School of Medicine, Yokohama City University, Yokohama, Japan

**Keywords:** liver transplantation, duct-to-duct anastomosis, biliary anastomotic stricture, blood perfusion of the extrahepatic bile duct, peribiliary vascular plexus, the 3 and 9 o’clock arteries

## Abstract

A duct-to-duct-biliary-anastomosis is the preferred biliary reconstruction technique in liver transplantation; biliary complications remain the major concerns for the technique. We examined the significance of the intramural vascular network of the extrahepatic bile duct (EBD) and its relevant vessels. We microscopically examined the axial sections of the EBD with 5 mm intervals of 10 formalin-fixed deceased livers. The luminal-areas of the 3 and 9 o’clock arteries correlated significantly and positively with the distance from the bifurcation of the right and left hepatic ducts (the 3 o’clock artery, *r* = 0.42, *p* < 0.001; the 9 o’clock artery, *r* = 0.39, *p* < 0.001); the ratios of the numbers of the intramural vessels to the areas of the corresponding sections of the EBD significantly correlated positively with the distance from the bifurcation of the right and left hepatic ducts (total vessels, *r* = 0.78, *p* < 0.001; arterioles, *r* = 0.52, *p* < 0.001; venules, *r* = 0.45, *p* < 0.001). This study demonstrated that there is a significant locoregional distributional heterogeneity of the intramural vessels among the EBD. The hepatic arteries neighboring the EBD primarily supply the blood flow to the EBD; thus, when the broader isolation of the EBD from the neighboring arteries is necessary, this locoregional distributional heterogeneity of the intramural vessels may render the EBD likely to suffer ischemia of the anastomotic site.

## Graphical Abstract

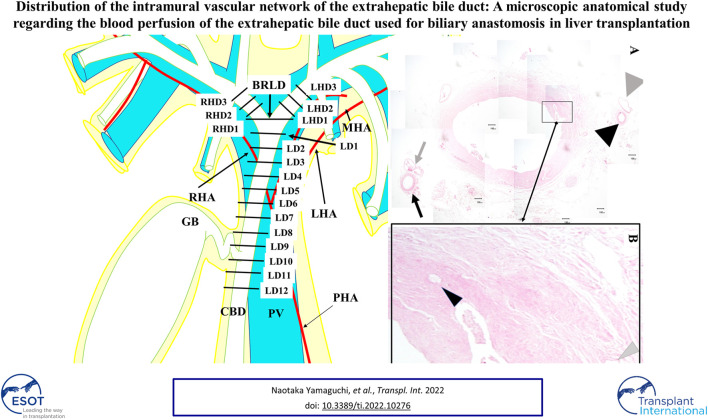

## Introduction

A duct-to-duct-biliary-anastomosis (DDBA) is the preferred biliary reconstruction technique in liver transplantation (LT); biliary-complications (BCs) remain the major concern with this technique (1–5). The blood perfusion of the extrahepatic bile duct (EBD) of the graft liver or the recipient is believed to affect the BCs strongly. The EBD is perfused by the peribiliary vascular plexus that consists of the following three layers: the inner-, intermediate-, and outer-layers (6).

Among these layers, the outer-layer corresponds to the connective tissue sheath surrounding the EBD; this sheath includes the abundant vascular network (6, 7). The outer-layer has been considered to act as the primary resource of the blood perfusion of the EBD; the arteries neighboring the EBD provide the blood inflow via the numerous thin macroscopically invisible arterioles into the EBD through the outer-layer (6–9).

Meanwhile, the intermediate-layer is regarded as the EBD itself. The EBD itself only has scarce intramural vessels; thus, the intramural vessels are considered insignificant for the blood perfusion of the EBD (6).

The unfavorable blood perfusion of the EBD of the graft liver or the recipient has been considered as the primary cause of the BCs; the significance of the outer-layer and the neighboring arteries has been vigorously discussed (1–5). This discussion argued that the hepatic arteries neighboring the EBD should be left as attached to the EBD as possible and the outer-layer should be as preserved as possible. However, the neighboring arteries often have to be isolated from the EBD to some extent to enable tension-free secure arterial and biliary anastomoses; this isolation leaves the outer-layer as the sole blood perfusion resource to the anastomotic site of the EBD other than the intramural-vessels. The incidence rate of the BCs remains high, at 20–50% (1–5). These findings may suggest that the preservation of the outer-layer alone cannot guarantee the favorable blood perfusion of the EBD of either the graft liver or the recipient (3–5); some additional insight into the blood circulation of the EBD may be necessary to reduce the incidence of the BCs.

Obtaining the overview of the intramural vessels of the EBD may lead to additional insight into the blood circulation of the EBD of the graft liver or the LT recipient. In this study, we investigated the intramural vascular network of the EBD using 10 deceased Japanese bodies.

## Materials and Methods

This study used 10 formalin-fixed adult Japanese deceased livers that had neither a history of previous hepatobiliary surgery nor any hepatobiliary diseases. The Institutional Review Board (IRB) of Yokohama City University approved and regarded this study to be compliant with the Hospital and the Declaration of Istanbul 2008 (IRB approve No. was #B19-1003045).

### Existence of the 3 and 9 O’Clock Arteries and Veins and Their Luminal Areas

**FIGURE 1:**
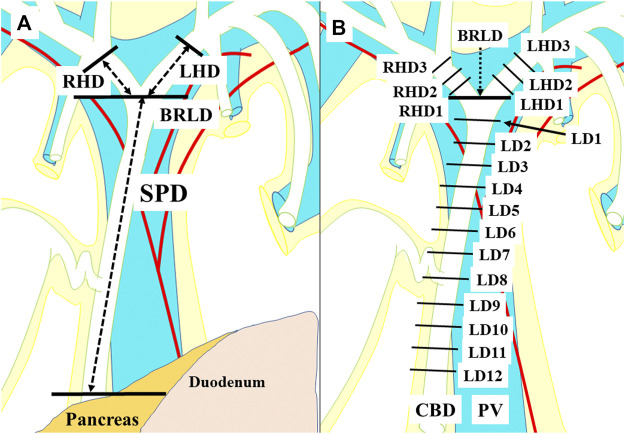
A term for each section for the histological examinations. The suprapancreatic duct was defined as the portion between the upper border of the pancreas and the bifurcation of the right and left hepatic ducts (BRLD) **(A)**. The ducts above the BRLD were the right hepatic duct (RHD) and the left hepatic duct (LHD) **(A)**. Axial sections with 5 mm intervals of the extrahepatic bile duct were prepared. Each section above the BRLD was termed LHD1, LHD2, and LHD3 along with the LHD, and RHD1, RHD2, and RHD3 along with the RHD from distal to proximal. The sections below the CRLD were termed CRLD, LD1, LD2, LD3, LD4, LD5, LD6, LD7, LD8, LD9, LD10, LD11, and LD12 from proximal to distal, respectively **(B)**.

We investigated the EBD above the upper margin of the pancreas in this study: the suprapancreatic duct, the left hepatic duct (LHD), and the right hepatic duct (RHD) (Figure 1A). First, the connective tissue was carefully dissected so as to preserve the peribiliary vascular plexus; this careful dissection enabled subsequent microscopical investigation of the 3 and 9 o’clock vessels. Then, axial sections of the EBD with 5 mm intervals were prepared. Each section was termed as shown in Figure 1B. Several 3-μm slices were excised from each surface of the sections. These slices were histologically examined after hematoxylin eosin staining.

**FIGURE 2:**
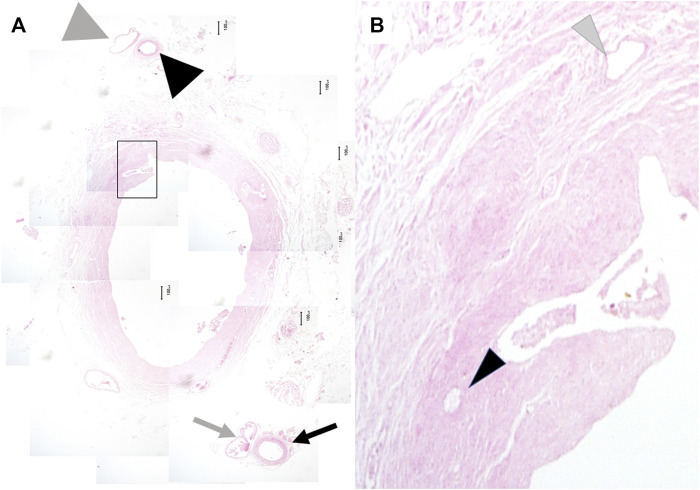
Microscopic findings of the extrahepatic bile duct. The 3 o’clock artery (black arrow) and vein (gray arrow) and the 9 o’clock artery (big black arrowhead) and vein (big gray arrowhead) were observed in all cases. The former was located immediately inside the right lateral border of the outer layer of the peribiliary vascular plexus. The latter was located immediately inside the right lateral border of the peribiliary vascular plexus **(A)** (original magnification, x40). Numbers of the arterioles (small black arrowhead) and venules (small gray arrowhead) were counted under the high-power field **(B)** (original magnification, x200).

Subsequently, we examined whether the 3 and 9 o’clock arteries and veins existed in the outer-layer of the peribiliary vascular plexus in each section (Figure 2A). If existed, the luminal areas of the 3 and 9 o’clock arteries and veins were calculated. In this study, the area microscopically evaluated was determined as follows. First, a microscopic field was projected onto a digitizing board; then, an object, of which we attempted to calculate the area, was outlined by a computerized delineation. Subsequently, the area of the object was calculated using Image J software (National Institute of Health, Bethesda, MD, United States) (8, 9). Then, these areas were compared among the sections; furthermore, their correlations with the distance from the bifurcation of the right and left hepatic ducts were investigated.

### Numbers of the Intramural Vessels of the Extrahepatic Bile Duct

At each section, the numbers of total intramural vessels, arterioles, and venules of the EBD were counted under high-power field of the microscopy (×200 magnification) (Figure 2B). Moreover, the area of the EBD wall in each section was determined; then, the ratios of the number of the intramural vessels to the areas of the corresponding sections of the EBD wall (/mm^2^) were calculated These ratios reflect the enrichment of the intramural vessels. These ratios were compared among the sections; their correlations with the distance from the bifurcation of the right and left hepatic ducts were investigated.

### Statistical Analysis

Numerical variables were expressed as median (range) and compared using the Wilcoxon rank sum test for paired variables. Post-hoc analyses were performed by the Holm-Bonferroni method. Correlation coefficient (r) was assessed with the Spearman rank correlation coefficient. Two-tailed *p* < 0.05 was accepted as significant. All statistical analyses were carried out using the SPSS commercial statistic software version 23 (IBM, Armonk, NY, United States).

## Results

### Existence of the 3 and 9 O’Clock Arteries and Veins

The outer-layer included the 3 and 9 o’clock arteries and veins in all cases. The 3 o’clock and 9 o’clock vessels ascended along the left and right lateral border of the outer-layer, respectively; at the confluence of the right and left ducts, the 3 o’clock vessels ascended along the left lateral border of the LHD. The 9 o’clock vessels ascended along the RHD. In other words, both the 3 and 9 o’clock vessels accompanied the suprapancreatic duct; however, the LHD or RHD had only one. Instead, a mesh-like-structure composed of thin arterial branches connecting with the LHD and/or RHD that arose from the RHA, LHA, and MHA existed above the bifurcation of the left and right hepatic ducts (Figures 2A, B).

### Luminal-Areas of the 3 and 9 O’Clock Arteries

**FIGURE 3:**
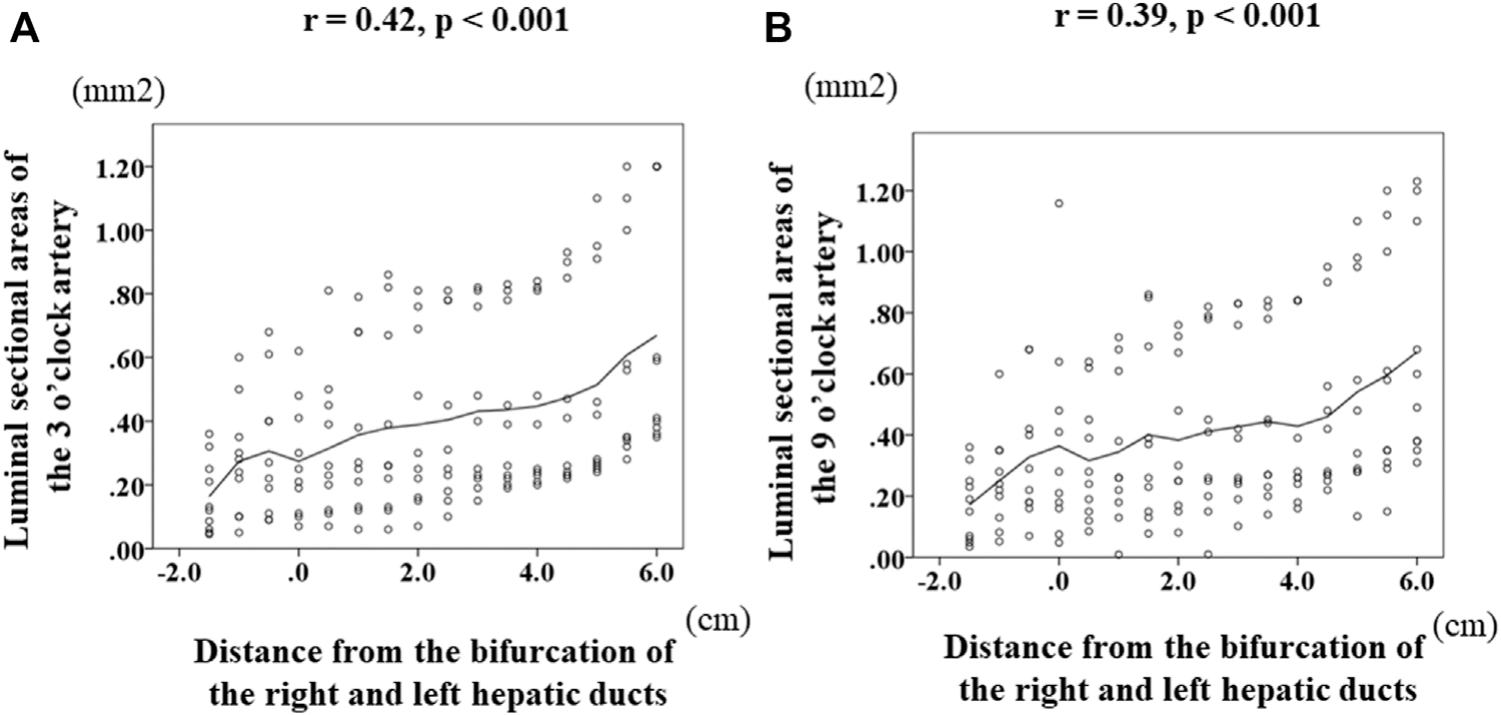
Transition of the luminal areas of the 3 and 9 o’clock arteries according to the distance from the bifurcation of the right and left hepatic ducts. Zero on the horizontal axis indicates the section at the bifurcation of the right and left ducts (BRLD). Luminal-area (mm^2^) of the 3 o’clock artery was 0.125 (0.045–0.36) at LHD3, 0.26 (0.05–0.6) at LHD2, 0.25 (0.09–0.68) at LHD1, 0.23 (0.07–0.62) at BRLD, 0.25 (0.07–0.81) at LD1, 0.26 (0.06–0.79) at LD2, 0.26 (0.06–0.86) at LD3, 0.8 (0.07–0.81) at LD4, 0.28 (0.10–0.81) at LD5, 0.33 (0.15–0.82) at LD6, 0.33 (0.20–0.84) at LD7, 0.32 (0.22–0.93) at LD8, 0.34 (0.24–1.10) at LD9, 0.35 (0.24–1.10) at LD10, 0.46 (0.28–1.20) at LD11, and 0.5 (0.35–1.20). A statistically significant difference was observed among the various combinations of these areas. Of note, the consecutive luminal areas of the 3 o’clock artery significantly correlated positively with the distance from the BRLD **(A)**. Regarding the 9 o’clock artery, the luminal areas were 0.17 (0.04–0.36) at RHD3, 0.23 (0.05–0.60) at RHD2, 0.26 (0.07–0.68) at RHD1, 0.25 (0.05–1.158) at CRLD, 0.26 (0.09–0.64) at LD1, 0.26 (0.01–0.72) at LD2, 0.32 (0.08–0.86) at LD3, 0.28 (0.08–0.76) at LD4, 0.34 (0.01–0.82) at LD5, 0.41 (0.10–2.25) at LD6, 0.36 (0.14–0.84) at LD7, 0.27 (0.16–0.84) at LD8, 0.35 (0.22–0.95) at LD9, 0.41 (0.13–1.10) at LD10, 0.47 (0.15–1.20) at LD11, and 0.55 (0.31–1.23) at LD12. A statistically significant difference was observed among the various combinations of these areas. Moreover, the consecutive luminal areas of the 9 o’clock artery correlated significantly and positively with the distance from the BRLD **(B)**.

Change of the luminal-area (mm^2^) of the 3 o’clock artery according to the sections was demonstrated in Figure 3A. A statistically significant difference was observed among the various combinations of the sections; the luminal area was smallest at the level of the bifurcation of the right and left hepatic ducts among these sections. Of note, the consecutive luminal areas of the corresponding sections of the 3 o’clock artery correlated significantly and positively with the distance from the bifurcation of the right and left hepatic ducts (*r* = 0.42, *p* < 0.001). Regarding the 9 o’clock artery, the change of the luminal areas was shown in Figure 3B. A statistically significant difference was observed among the various combinations of these sections. Moreover, the consecutive luminal areas of the corresponding sections of the 9 o’clock artery significantly correlated positively with the distance from the bifurcation of the right and left hepatic ducts (*r* = 0.39, *p* = < 0.001).

### The Ratios of the Numbers of the Intramural Vessels to the Areas of the Corresponding Sections of the Extrahepatic Bile Duct

**FIGURE 4:**
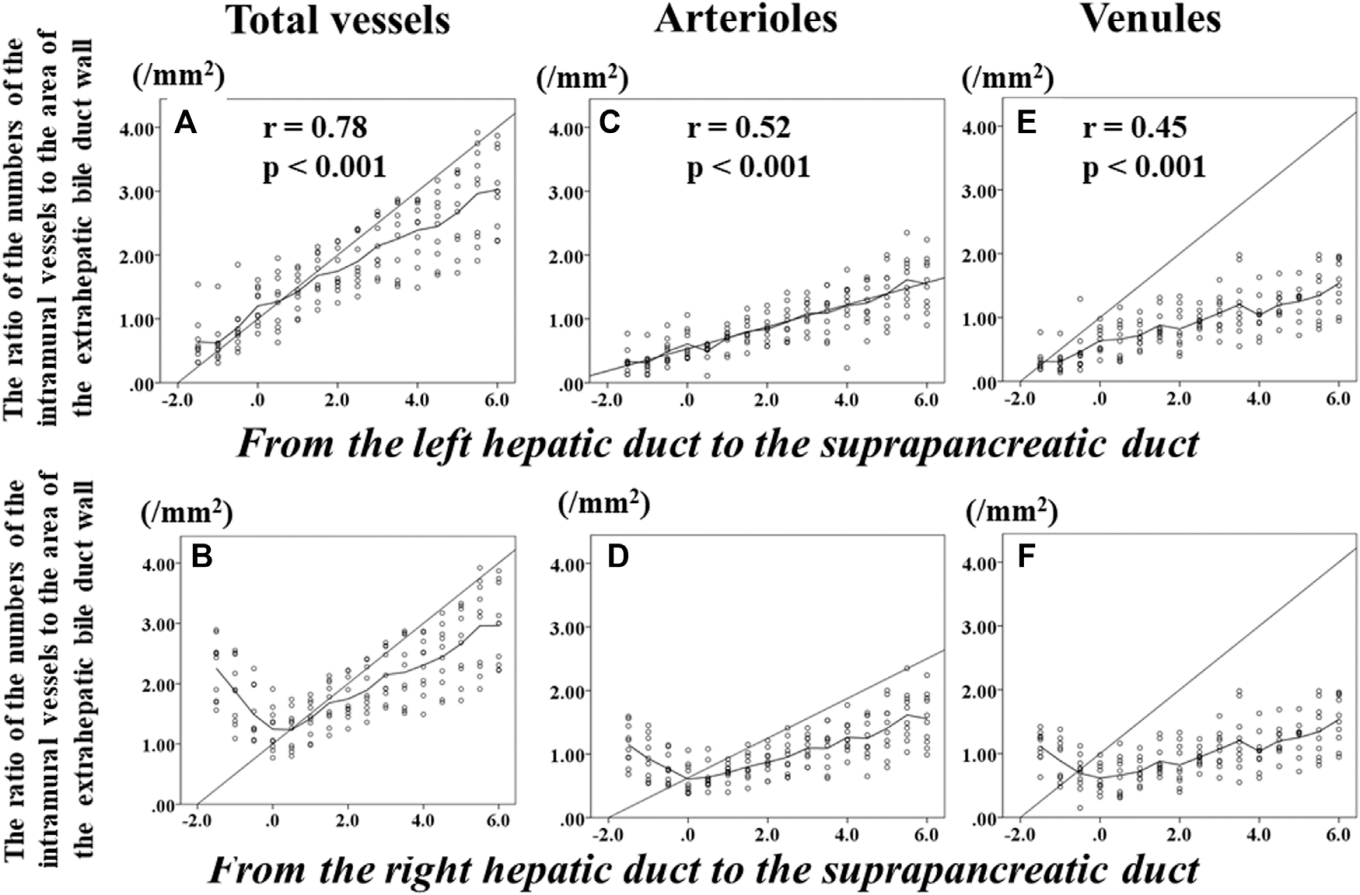
Transition of the numbers of total intramural vessels, arterioles, and venules of the extrahepatic bile duct according to the distance from the bifurcation of the right and left hepatic duct. Zero on the horizontal axis indicates the section at the bifurcation of the right and left hepatic ducts (BRLD). Upper left **(A)** shows the changes in the ratios of the numbers of total intramural vessels to the area of the corresponding sections of the extrahepatic bile duct (EBD) from the left hepatic duct (LHD) to the suprapancreatic duct (SPD). Lower left **(B)** indicates alteration of the ratios of the numbers of total intramural vessels of the EBD from the right hepatic duct (RHD) to the SPD. Beyond the BRLD, the numbers of total intramural vessels of the LHD increased and those of the RHD decreased when getting closer to the BRLD. Below the BRLD, however, the ratios of the numbers of total intramural vessels of the EBD increased as the distance from the BRLD increased. Although a difference in the transition of the ratios of the numbers of total intramural vessels to the areas of the corresponding areas of the EBD was observed between the LHD and the RHD, the ratio of the numbers of total intramural vessels of total intramural vessel of the EBD continued to rise as the distance from the BRLD increased in the SPD. Of note, the ratio of total intramural vessels to the area of the corresponding area at the BRLD [1.21 (0.77–1.91)] was significantly smaller than at the LD2 [1.43 (0.98–1.82), *p* = 0.022], LD3 [1.66 (1.14–2.13), *p* = 0.005], LD4 [1.61 (1.25–2.22), *p* = 0.005], LD5 [1.78 (1.36–2.41), *p* = 0.005], LD6 [2.03 (1.60–2.68), *p* = 0.005], LD7 [2.14 (1.51–2.87), *p* = 0.005], LD8 [2.40 (1.49–2.87, *p* = 0.005], LD9 [2.52 (1.69–3.17), *p* = 0.005], LD10 [2.54 (1.72–3.33), 0.005], LD11 [3.15 (1.90–3.92), *p* = 0.005], and LD12 [3.00 (2.22–3.87), *p* = 0.005], respectively. In addition, below the BRLD, the numbers of total intramural vessels of the EBD significantly correlated positively with the distance from the BRLD. When the intramural arterioles and venules were analyzed separately, significant differences of the ratios of the numbers of intramural arterioles or venules between the BRLD and several points below the BRLD were observed. In addition, below the BRLD, the statistically significant correlation among the numbers per unit area of either arteriole or venules and the distance from the BRLD was observed **(C–F)** (arterioles, *r* = 0.515, *p* < 0.001; venules, *r* = 0.449, *p* < 0.001). These results indicate that below the BRLD, the more distal the location to the distance from the BRLD, the higher the ratios of the numbers of intramural total vessel, arteriole, and venules of the EBD.

Figure 4 shows the changes in the ratio of the numbers of total intramural vessels to the areas of the corresponding sections of the EBD according to the distance from the confluence of the right and left hepatic ducts (0 of the horizontal axis label indicates the level of the confluence of the right and left hepatic ducts); this indicates the changes in the ratios from the LHD to the suprapancreatic duct. Figure 4B indicates the alteration of the ratios from the RHD to the suprapancreatic duct. Beyond the bifurcation of the right and left hepatic ducts, the numbers of total intramural vessels of the LHD (Figure 4A) increased and those of the RHD (Figure 4B) decreased as the corresponding sections were getting closer to the bifurcation of the right and left hepatic ducts. Beneath the bifurcation of the right and left hepatic ducts, however, the higher the ratios of the numbers of total intramural vessels to the areas of corresponding sections of the EBD, the further the distance from the bifurcation of the right and left ducts (Figures 4A,B). Above the bifurcation of the right and left hepatic ducts, there was no correlation between the ratios of the numbers of vessels to the areas of the corresponding section of the LHD or RHD and the distance from the bifurcation of the right and left hepatic ducts; the ratios of the number of the intramural -vessels to the areas of the corresponding sections of the suprapancreatic duct continued to rise as the distance from the bifurcation of the right and left hepatic ducts increased. Of note, the ratio of the numbers of total intramural vessels to the area of the EBD wall at the bifurcation of the right and left hepatic ducts was fewest among the sections. In addition, below the bifurcation of the right and left hepatic ducts, the ratio of the number of total intramural vessels to the areas of the corresponding sections significantly correlated positively with the distance from the bifurcation of the right and left hepatic ducts (*r* = 0.78, *p* < 0.001) (Figures 4A,B).

When the arterioles and venules were analyzed separately, the significant differences of the ratios of the number of each vessel to the area of the corresponding sections were observed between the bifurcation of the right and left hepatic ducts and several points beneath the bifurcation of the right and left hepatic ducts (Figures 4C–F). In addition, the ratios of the numbers of the intramural arterioles and venules to the areas of the corresponding sections correlated significantly and positively with the distance from the bifurcation of the right and left hepatic ducts in the suprapancreatic duct (arterioles, *r* = 0.52, *p* < 0.001; venules, *r* = 0.45, *p* < 0.001).

## Discussion

The most important of our findings is that there is a significant locoregional heterogeneity of the numbers and luminal broadness of the intramural vessels among the EBD. If the neighboring arteries are left attached to the EBD, this heterogeneity may be not significant because of the numerous bridging arterioles connecting the neighboring arteries to the EBD. A few institutions argued that the dissection between the neighboring arteries and the EBD must be minimal to retain the sufficient blood flow to the EBD and this procedure can almost always be performed (3–5). However, the hepatic arteries and the EBD often need to be isolated from each other for elongation that enables the tension-free secure reconstruction (1, 2, 10, 11); this isolation requires the dissection of the hepatic arteries from the EBD that unavoidably divide the connecting arterioles between the hepatic arteries and the EBD. On such occasion, the locoregional heterogeneity of the intramural vessels may impair the perfusion of the EBD. This is especially true in the graft liver EBD of the deceased donor LT (DDLT), where the blood flow goes through the proximal EBD to reach the distal end of the EBD; namely, blood flow passes through the thinner and fewer intramural vessels and reaches the thicker and more abundant intramural vessels. This means that the amount of blood flow bdecreases for the sizes of the intramural vessels as it goes further; thus, this is likely to cause the ischemia of the anastomotic site of the graft liver EBD. The ischemia may deteriorate as the length of the EBD isolated from the hepatic arteries grows. The deterioration of the ischemia easily causes the BCs, including the anastomotic leakage, anastomotic stricture, and the ischemic cholangiopathy (1–5, 10, 11), In the recipient of the living donor LT (LDLT) using DDBA, the vicinity of the bifurcation of the right and left hepatic ducts of the EBD is usually used for the anastomotic site. Our present findings showed that the intramural vessels are thinnest and fewest in the bifurcation of the right and left hepatic ducts; this suggests that the scarcity of the intramural vessels may cause the blood flow paucity at the anastomotic site of the recipient EBD, especially when the EBD is isolated extensively from the neighboring hepatic arteries. With regard to the DDLT recipient, the distributional imbalance of the intramural vessels between the proximal and distal parts of the EBD is likely to become less severe compared to the LDLT recipient because the EBD is divided more distally in the DDLT than in the LDLT. However, the location of the graft EBD to anastomose will be strongly associated with the decision of the anastomotic site of the recipient EBD; if the graft EBD is divided at an unusually proximal site, it requires further isolation of the EBD from the neighboring arteries to elongate the recipient EBD to achieve the tension-free secure biliary anastomosis. As a result, the anastomotic site of the recipient EBD becomes likely to suffer the ischemia.

As such, the anastomotic site of both the graft and recipient EBD is likely to suffer the ischemia because of the locoregional distributional heterogeneity of the intramural vessels. We consider that it requires some technical ingenuity to avoid this ischemia which is inevitable on some occasions. One solution may be a more distal implantation of the graft livers compared to the orthotopic implantation (10, 11); this enables the tension-free biliary anastomosis even in occasions where both the graft and the recipient EBDs are short for the tension-free anastomosis in the orthotopic implantation.

There are several limitations in this study. First, we used the livers of deceased bodies; this study cannot assess the actual hemodynamics. To confirm the significance of our present findings in a clinical setting, we are currently performing a study that can assess the actual hemodynamics of the EBD by the near-infrared imaging using indocyanine green (12). Second is the exclusion of the donors who had a history of previous hepatobiliary surgery and/or any hepatobiliary diseases. The LT recipients have severe hepatobiliary diseases and a certain population of them received some hepatobiliary surgery; these pathologic conditions undoubtedly affect the blood-circulation of the recipient EBD. The exclusion of the donors without such pathologic conditions rendered this study unsuitable for obtaining an insight into the recipient EBD. However, the inclusion of the donors who had such conditions leads to the enormous heterogeneity of the study samples; this heterogeneity requires an extremely large sample size to neutralize. Unfortunately, we had a limited number of the donors. Thus, we had to limit the donors who had neither a history of previous hepatobiliary surgery and/or any hepatobiliary diseases to this study sample to counteract the heterogeneity as much as possible. Meanwhile, the present study was quite suitable for gaining insight into the perfusion of the EBD of the graft liver, especially the DDLT graft. Third, the anatomy of the biliary system and surrounding vascular network vary markedly (6–9). Therefore, our sample size of 10 deceased Japanese bodies may be too small to draw any conclusion. However, this study focused specifically on the intramural vascular network of the suprapancreatic duct; the anatomy, its feeding arteries, and its drainage veins of this part were reported to be less various compared to the other part (6, 7). Thus, a sample size of 10 may be sufficient for analyzing the intramural vascular network of the suprapancreatic duct; in fact, the variables we examined were quite less diverse. However, we realize that the results of the present study were debatable because of the probable bias due to the small sample size. Besides, we recognize that the hepatobiliary diseases from which the liver transplant recipients suffer easily cause the structural and/or functional alterations of the EBD; these alterations have to be taken into consideration. Therefore, we will again examine the issues discussed in the present study in future studies using larger samples that include donors who have a history of previous hepatobiliary surgery and/or hepatobiliary diseases. Despite these limitations, we believe that this study is valuable because this is the first report focusing on the intramural vascular network of the EBD relevant to the DDBA in either the DDLT or the LDLT.

In conclusion, our present findings demonstrated that there is a significant distributional imbalance of the intramural vessels between the proximal and distal parts of the EBD; this could have a significant negative impact on the perfusion of the EBD, especially when the EBD has to be broadly isolated from the neighboring arteries. When the broader isolation of the EBD from the neighboring arteries is necessary, it requires some technical ingenuity for the DDBA to avoid the ischemia of the anastomotic site of the EBD; this needs to take the locoregional heterogeneity of the intramural vessels into consideration.

## Data Availability

The raw data supporting the conclusion of this article will be made available by the authors, without undue reservation.
